# Comparative genomic analysis of two Arctic *Pseudomonas* strains reveals insights into the aerobic denitrification in cold environments

**DOI:** 10.1186/s12864-023-09638-1

**Published:** 2023-09-11

**Authors:** Yong-Qiang Hu, Yin-Xin Zeng, Yu Du, Wei Zhao, Hui-Rong Li, Wei Han, Ting Hu, Wei Luo

**Affiliations:** 1 Key Laboratory for Polar Science, Polar Research Institute of China, Ministry of Natural Resources, Shanghai, 200136 China; 2 School of Oceanography, Shanghai Jiao Tong University, Shanghai, 200030 China

**Keywords:** *Pseudomonas*, Arctic, Aerobic denitrification, Cold adaptation, Heavy metal resistance

## Abstract

**Background:**

Biological denitrification has been commonly adopted for the removal of nitrogen from sewage effluents. However, due to the low temperature during winter, microorganisms in the wastewater biological treatment unit usually encounter problems such as slow cell growth and low enzymatic efficiency. Hence, the isolation and screening of cold-tolerant aerobic denitrifying bacteria (ADB) have recently drawn attention. In our previous study, two *Pseudomonas* strains PMCC200344 and PMCC200367 isolated from Arctic soil demonstrated strong denitrification ability at low temperatures. The two Arctic strains show potential for biological nitrogen removal from sewage in cold environments. However, the genome sequences of these two organisms have not been reported thus far.

**Results:**

Here, the basic characteristics and genetic diversity of strains PMCC200344 and PMCC200367 were described, together with the complete genomes and comparative genomic results. The genome of *Pseudomonas* sp. PMCC200344 was composed of a circular chromosome of 6,478,166 bp with a G + C content of 58.60% and contained a total of 5,853 genes. The genome of *Pseudomonas* sp. PMCC200367 was composed of a circular chromosome of 6,360,061 bp with a G + C content of 58.68% and contained 5,801 genes. Not only prophages but also genomic islands were identified in the two *Pseudomonas* strains. No plasmids were observed. All genes of a complete set of denitrification pathways as well as various putative cold adaptation and heavy metal resistance genes in the genomes were identified and analyzed. These genes were usually detected on genomic islands in bacterial genomes.

**Conclusions:**

These analytical results provide insights into the genomic basis of microbial denitrification in cold environments, indicating the potential of Arctic *Pseudomonas* strains in nitrogen removal from sewage effluents at low temperatures.

**Supplementary Information:**

The online version contains supplementary material available at 10.1186/s12864-023-09638-1.

## Background

Discharge of sewage with a large amount of nitrogen to a body of water may result in public health hazards. The problem can be mitigated by eliminating nitrogen from sewage before it is discharged. Nitrogen in sewage can exist in four forms, including organic nitrogen, ammonia, nitrite and nitrate (https://nature.berkeley.edu/classes/es196/projects/2001final/Kurosu.pdf). Organic nitrogen and ammonia nitrogen are the principal forms in untreated sewage. They can be converted into nitrogen gas and thus removed from the water by using traditional biological nitrogen removal technology based on sequential nitrification and denitrification techniques [1]. However, the aerobic nitrification and anaerobic denitrification processes have completely contrary requirements for organic matter and oxygen and are sensitive to environmental factors and operational conditions. To solve the problems of complexity and treatment cost of the process caused by traditional biological denitrification, the nitrogen removal advantages of aerobic denitrifying bacteria (ADB) have recently become a hot spot for the scientific community [2–6]. Isolation and screening of ADB agents can enrich the database of aerobic denitrifying strains.

Temperature affects the denitrification efficiency by affecting the cell growth and enzyme activity of ADB [1]. Most aerobic denitrification reactions occur at medium temperatures between 20 °C and 40 °C. Thus, cold-tolerant ADB have the potential for effective nitrogen removal in winter and can substantially reduce operating costs. A few cold-tolerant ADB, including *Rhizobium* sp. WS7 [7], *Acinetobacter* sp. TAC-1 [8], *Pseudomonas putida* Y-9 [9] and *P. psychrophilia* RNC-1 [10], have been isolated from various environments. However, the denitrification mechanism of those ADB at the molecular level remains poorly understood. The psychrophilic and psychrotrophic microorganisms that inhabit the polar regions on Earth have important applications in bioremediation, medicine, pharmaceuticals and many other areas of biotechnology [11]. These microorganisms are also a source of ADB with high denitrification efficiency at low temperatures.

Two *Pseudomonas* strains, PMCC200344 (previously known as S012-3) and PMCC200367 (previously known as S025), were isolated from soils under colorful snow in Arctic Ny-Alesund, Svalbard. The two bacterial strains not only grew well between 4 °C and 25 °C but also showed aerobic denitrifying ability growing on bromthymol blue (BTB) medium plate at 10 °C. The BTB medium used for isolating and screening aerobic denitrifiers was proposed by Takaya et al. [12]. Because of these characteristics, the two strains have strong application potential for removing nitrogen from sewage in cold environments. However, the genome sequence and basic properties of the two bacteria have not been reported thus far. Therefore, the complete genomic information of strains PMCC200344 and PMCC200367 was reported, and comparative genomic analysis with other relevant sequenced reference genomes was performed in the present study.

## Results

### Organism characteristics and classification

**Fig. 1:**
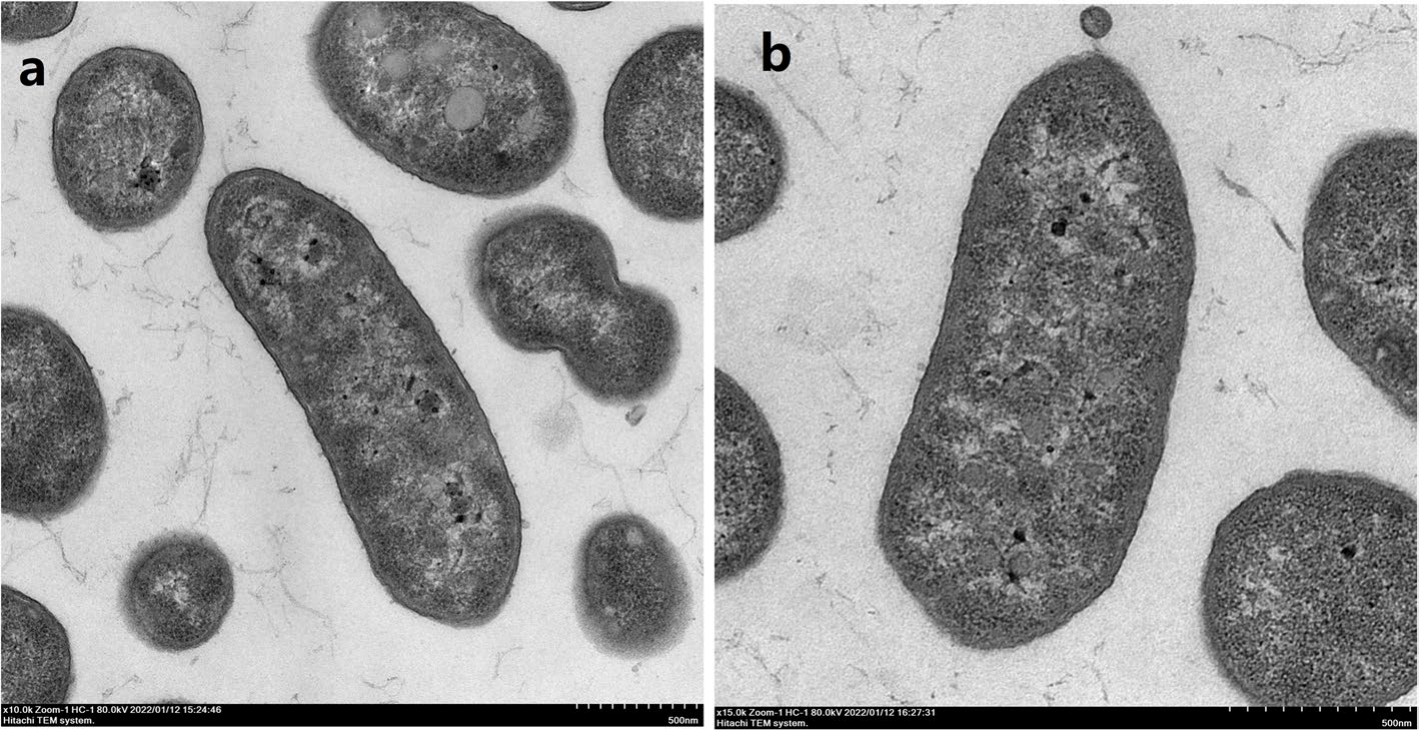
Transmission electron micrographs of the cellular morphology of *Pseudomonas* strains PMCC200344 (**a**) and PMCC200367 (**b**)

Colonies of strains PMCC200344 and PMCC200367 grown on R2A agar plates were cream-colored, uniformly round, convex, smooth, moist and opaque with entire margins. Additionally, R2A agar plates were stained yellow due to the growth of the bacteria. Cells of both strains were Gram-stain-negative, aerobic, motile, non-spore-forming and rod-shaped (Fig. 1). Growth of the two strains occurred at 1–34 ℃, pH 5.5–9.0, and 0–6% NaCl. The basic characteristics and classification of strains PMCC200344 and PMCC200367 are shown in Table S1. API 20 NE analysis demonstrated that the two strains could denitrify nitrate to nitrogen gas.

Colonies of the two bacterial strains grown on BTB medium plates were found to be blue, suggesting that they are aerobic denitrifiers [12]. Denitrifying activity assays revealed that, after 48 h cultivating at 10 °C, the nitrate removal efficiency and total nitrogen removal efficiency of strain PMCC200344 in denitrifying medium (DM) reached 78.9% and 67.9%, respectively, without nitrite accumulation. In contrast, nitrate removal efficiency and total nitrogen removal efficiency of strain PMCC200367 reached 90.3% and 74.5%, respectively, with low accumulation of nitrite as an intermediate in the process of denitrification.

**Fig. 2:**
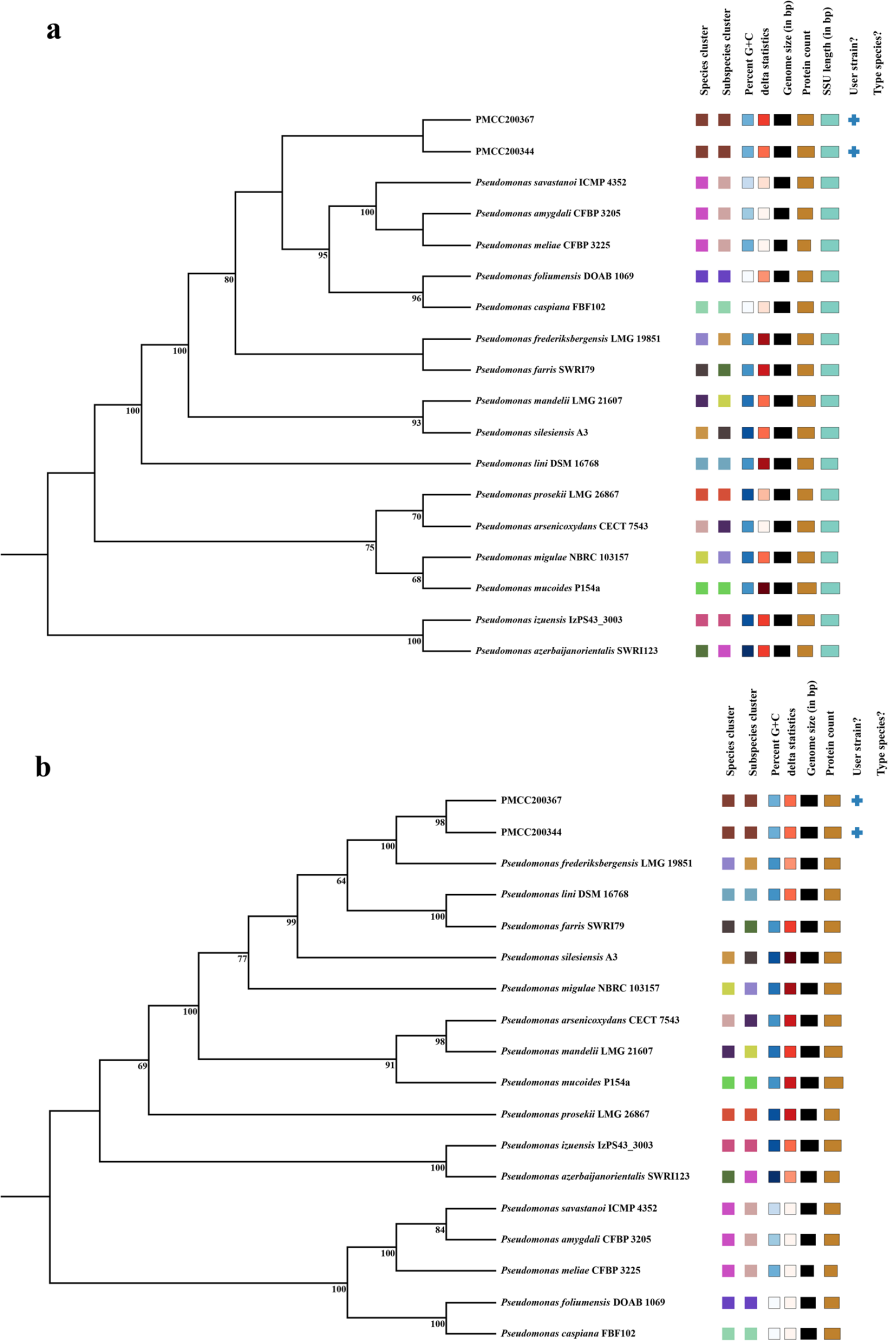
Genome BLAST Distance Phylogeny method (GBDP) for phylogenetic placement analysis using FastME 2.1.6.1 with 100 bootstrap values. **a** 16S rRNA gene sequence-based phylogeny of strains PMCC200344 and PMCC200367 with the closely related type strains and whole genomes with 77.3% average branch support. **b** Whole-genome sequence-based phylogeny of strains PMCC200344 and PMCC200367 with the closely related type strains and whole genomes with 92.0% average branch support. The numbers above branches represent the GBDP pseudobootstrap value greater than 60%

According to the EzBioCloud server analysis, strains PMCC200344 and PMCC200367 exhibited close phylogenetic affinity to *Pseudomonas silesiensi* A3^T^, *P. mandelii* NBRC103147^T^ and *P. frederiksbergensis* JAJ28^T^ with sequence similarity values higher than 99.50%. The phylogenetic tree inferred from the intergenomic distance calculated from Genome BLAST Distance Phylogeny (GBDP) in the Type Strain Genome Server (TYGS) is shown in Fig. 2. The results indicate that strains PMCC200344 and PMCC200367 are close to each other and are the closest relative to *P. frederiksbergensis* LMG 19851^T^ (= JAJ28^T^). The average nucleotide identity (ANI) was determined based on the whole-genome data for bacterial species classification. An ANI value of 98.72% was detected between strains PMCC200344 and PMCC200367, indicating that the two strains are within the same *Pseudomonas* species. However, the ANI values of the two strains to the close relatives were all lower than 94% (Table S2), which is less than the species threshold value of 95% [13]. In addition, the digital DNA‒DNA hybridization (dDDH) value between strains PMCC200344 and PMCC200367 was as high as 91.3%, while the two strains showed only 62.5% and 63.4% similarity to *P. frederiksbergensis* LMG 19851^T^, respectively (Table S3), which were below the threshold value of 70% accepted for species delineation [14]. The results indicate that strains PMCC200344 and PMCC200367 represent a novel species of the *Pseudomonas* genus. More detailed results describing the two bacterial strains are considered to publish in another academic journal special for novel microbial taxa.

### Genome features

**Fig. 3:**
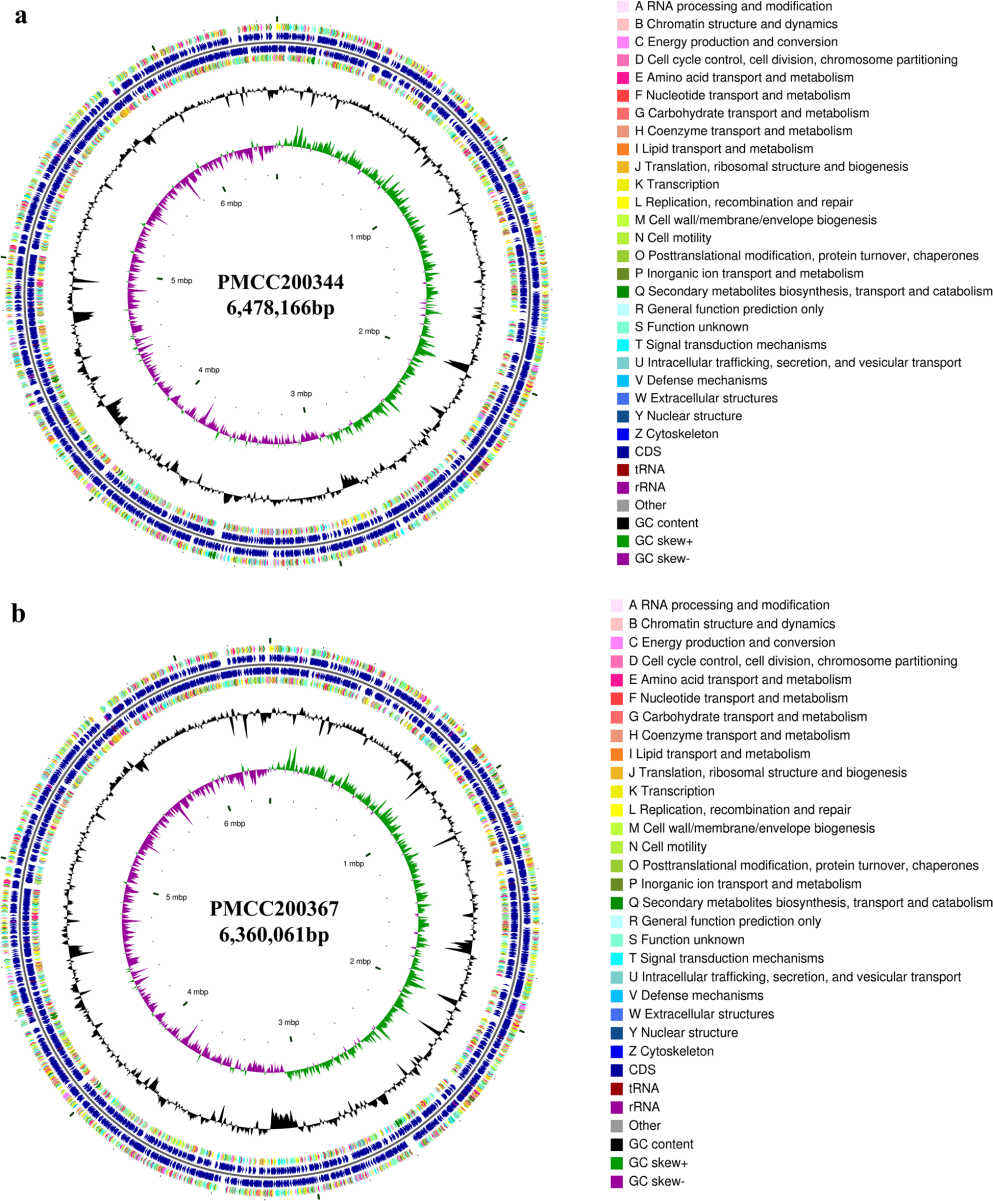
Circular maps of *Pseudomonas* sp*.* PMCC200344 (**a**) and PMCC200367 (**b**). From outside to center, rings 1 and 4 show protein-coding genes colored by COG categories on the forward/reverse strand; rings 2 and 3 denote genes on the forward/reverse strand; ring 5 shows the G + C % content plot; ring 6 shows the GC skew; the innermost ring shows the marker of genome size

**Fig. 4:**
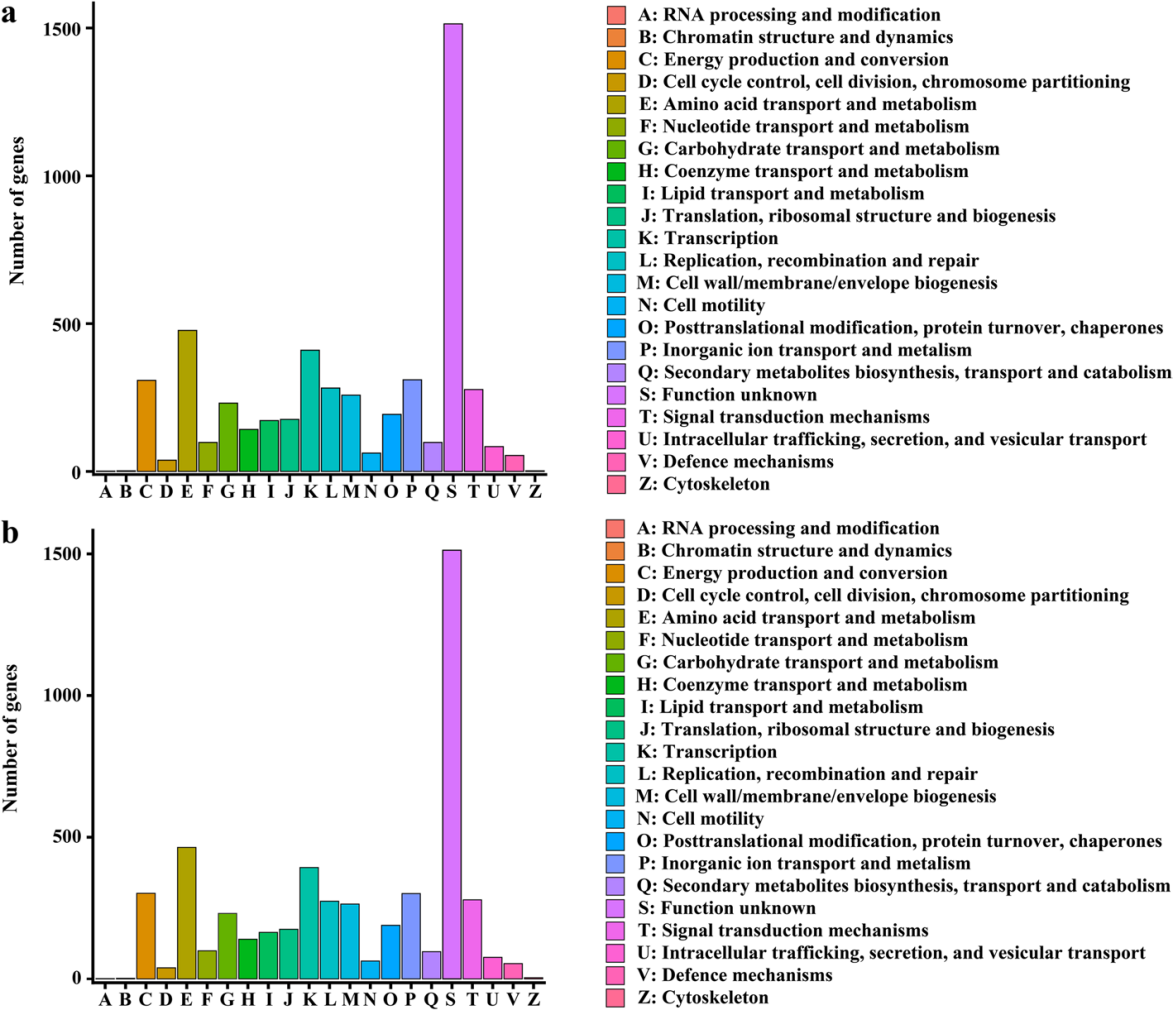
Number of genes associated with the 22 general COG functional categories in the genome of *Pseudomonas* sp. PMCC200344 (**a**) and PMCC200367 (**b**)

Genomic properties and statistics are shown in Table S4. Strain PMCC200344 contained a circular chromosome of 6,478,166 bp with a 58.60% G + C content (Fig. 3). A total of 5,853 genes, including 5,335 protein-encoding genes and 195 RNA genes (including 23 rRNA, 64 tRNA and 108 sRNA) were predicted on the chromosome of strain PMCC200344. The genome contained one prophage and 99 genomic islands. In addition, 5,099 genes (87.12%) were classified into 22 functional Clusters of Orthologous Groups (COG) categories (Fig. 4). Among them, the six most abundant genes were those with functions related to unknown (category S; 29.69%), amino acid transport and metabolism (category E; 9.35%), transcription (category K; 8.04%), inorganic ion transport and metabolism (category P; 6.08%), energy production and conversion (category C; 6.04%), and signal transduction mechanisms (category T; 5.43%).

Strain PMCC200367 contained a circular chromosome of 6,360,061 bp with a 58.68% G + C content. A total of 5,801 genes, including 5,286 protein-encoding genes and 200 RNA genes (including 23 rRNA, 65 tRNA and 112 sRNA) were predicted on the chromosome of strain PMCC200367. The genome of PMCC200367 contained two prophages and 102 genomic islands. In addition, 5,032 genes (86.59%) were distributed into COG categories. Among them, the six most abundant genes were those with functions related to unknown (category S; 30.07%), amino acid transport and metabolism (category E; 9.22%), transcription (category K; 7.81%), energy production and conversion (category C; 6.00%), inorganic ion transport and metabolism (category P; 5.98%), and signal transduction mechanisms (category T; 5.54%).

### Identification of the nitrate reduction pathway

**Fig. 5:**
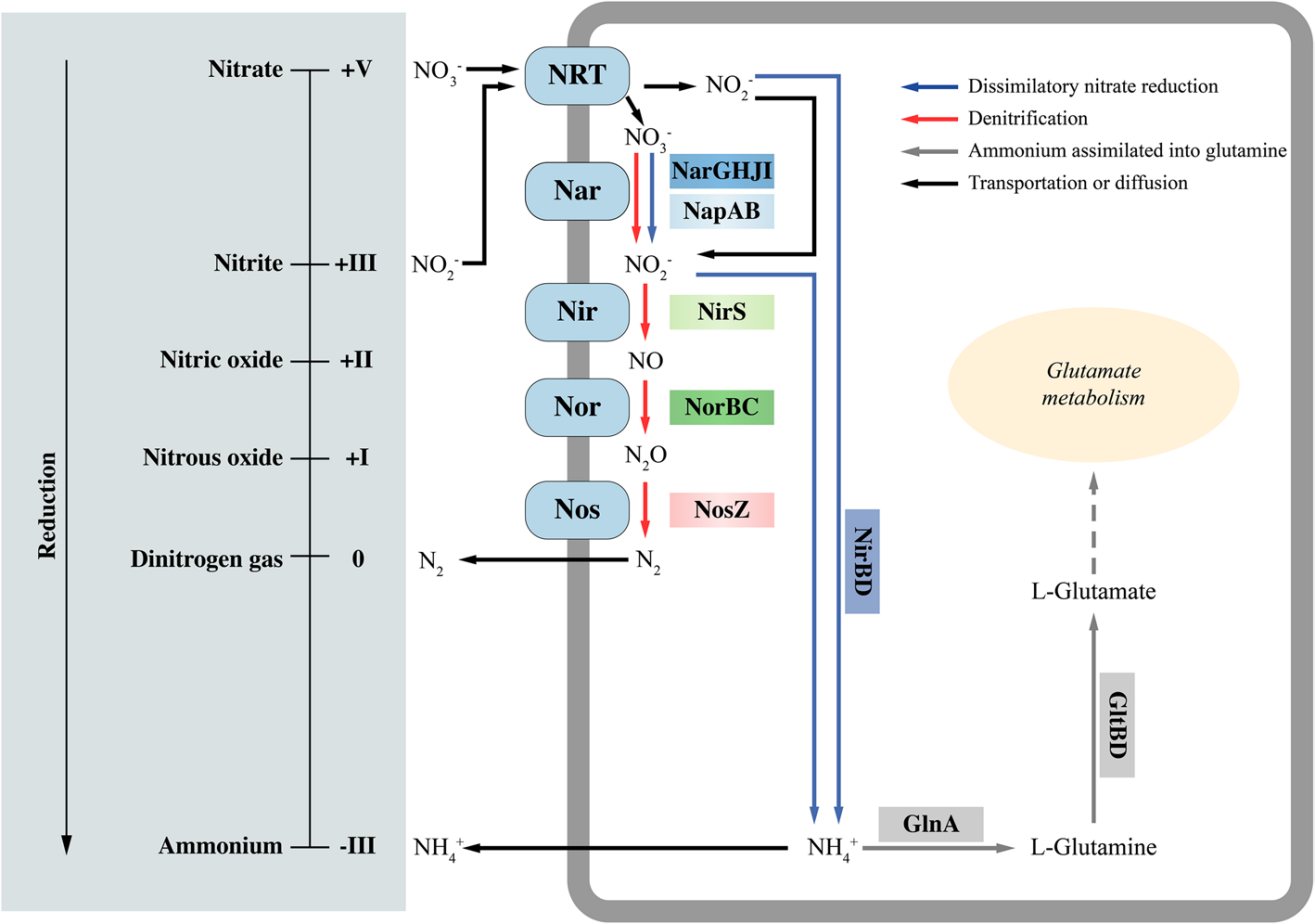
Putative nitrate reduction pathways and related genes in *Pseudomonas* strains PMCC200344 and PMCC200367 after KEGG annotation [15–17]

According to the Kyoto Encyclopedia of Genes and Genomes (KEGG) prediction analysis [15–17], both strains (PMCC200344 and PMCC200367) contained all genes of the complete set of denitrification pathways (Fig. 5), including *napA*, *napB*, *narG*, *narH*, *narI*, *nirS*, *norB*, *norC* and *nosZ*. The results provided the genome basis of the two strains with the ability to reduce nitrate to dinitrogen gas. Furthermore, genes related to the nitrate transport protein NarK and related regulatory proteins NarX and NarL were detected in the genomes of both strains (Fig. S1). In addition, the nitrate assimilation pathway was also detected in the genomic data of the two strains. For example, *nirBD* encoding nitrite reductase as well as *glnA* encoding glutamine synthetase were detected in the genomes (Fig. 5), indicating that inorganic nitrogen can be assimilated into organic matter by the two *Pseudomonas* strains. Basic information on the genes involved in nitrate reduction pathways, including gene ID on the chromosome, gene name and gene description, is shown in Table S5.

However, genes related to the ammonia oxidation pathway (e.g., *amoA* and *nxrB*) were not detected in the genome of both *Pseudomonas* strains, indicating that the two bacteria are not related to nitrification ability.

### Identification of cold adaptation and heavy metal resistance genes

Strains PMCC200344 and PMCC200367 were psychrotrophic (Table S1). According to the results of genome annotation, the two strains contained multiple putative functional proteins related to cold adaptation, including cold shock proteins (e.g., CspA), DEAD-box RNA helicases (e.g., DeaD), ribosome binding factor A (e.g., RbfA), trehalose biosynthesis enzymes (e.g., TreS), and proline/glycine betaine-binding proteins (e.g., ProX). The genes related to cold adaptation were randomly distributed in the genome.

**Fig. 6:**
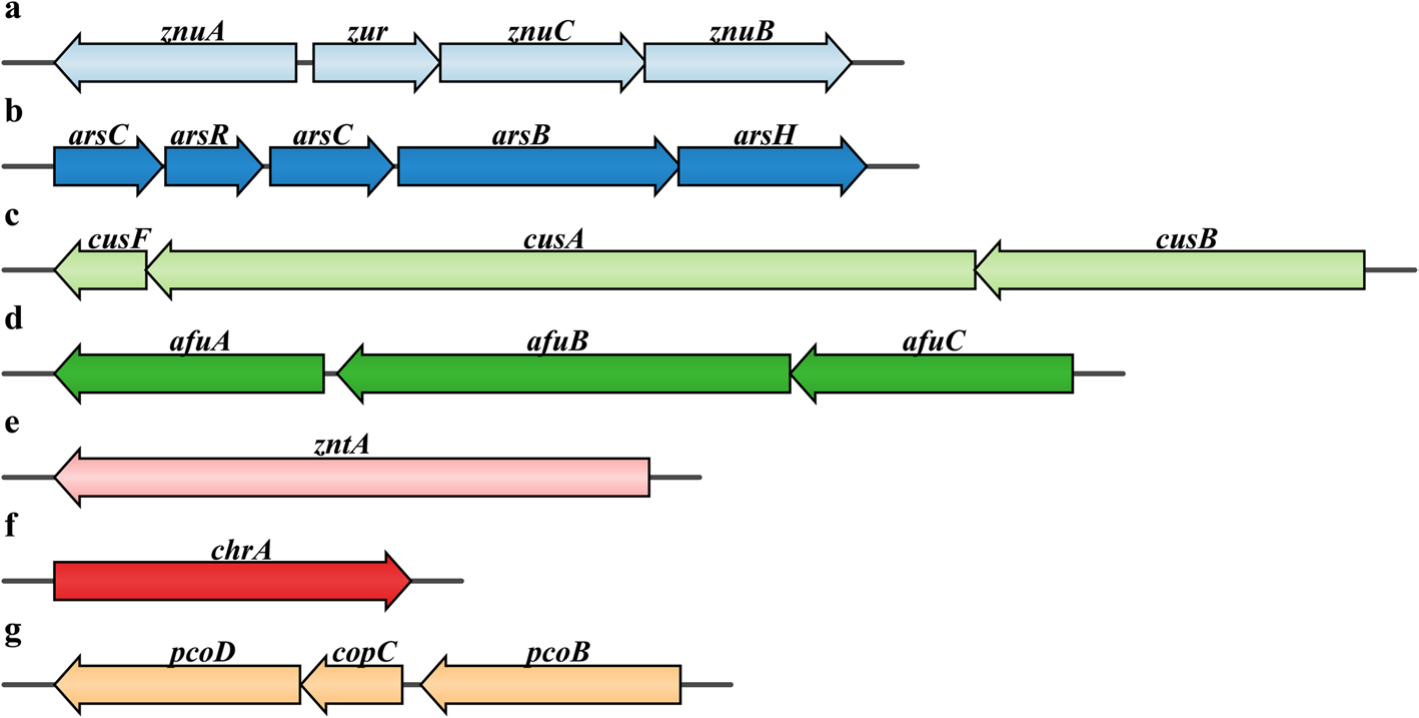
Heavy metal resistance genes distributed in PMCC200344 and PMCC200367. **a** Zinc ABC transporter permease, **b** arsenical resistance protein, **c** copper resistance system, **d** iron (III) transport system, **e** zinc/cadmium/lead-transporting ATPase, **f** chromate transporter, **g** copper resistance gene

Furthermore, multiple putative functional proteins related to heavy metal resistance, including transporters (e.g., chromate transporter), resistance proteins (e.g., copper resistance system) and metal reductases (e.g., arsenate reductase ArsC), were also observed in the genome of the two strains (Fig. 6). Many of those genes (e.g., *znuB*, *znuC* and *znuA*) were clustered in the genome. In addition, a large number of heavy metal resistance gene clusters were found to have multiple copies in the genomes of strains PMCC200344 and PMCC200367.

### Identification of genomic islands

The genomic islands in the PMCC200344 and PMCC200367 genomes were predicted by IslandViewer 4 combined with genome annotation, and all genes on the genomic islands were further analyzed (Tables S6 and S7). Complete denitrification pathway genes as well as many heavy metal resistance and cold adaptation genes were found to be present on the genomic islands in both bacteria (Table 1). In addition, the catalase gene *katE*, which encodes a key enzyme that protects bacteria from damage caused by reactive oxygen species (ROS), was also found on genomic islands. However, genes related to denitrification, heavy metal resistance, membrane transport and ROS resistance were usually not distributed on the same gene island.

**Table 1:** Genomic islands in the genomes of *Pseudomonas* sp*.* PMCC200344 and PMCC200367

Strain	Method	Start	End	Denitrification and resistance gene
PMCC200344	IslandPath-DIMOB, SIGI-HMM	4,144,457	4,231,195	*cusF*, *cusA*, *cusB*, *cusS*, *cusR*, *pcoD*, *copC*, *pcoB*, *copA*
	IslandPath-DIMOB, SIGI-HMM	6,433,467	6,467,413	*pcoD*, *copC*, *cusS*, *cusR*, *copA*, *copZ*, *pcoB*
	IslandPick	2,813,914	2,820,865	*arsC*, *arsR*
	IslandPick	3,236,432	3,253,340	*norD*, *norB*, *norC*, *norE*, *norQ*, *nirS*, *nirC*, *nirF*
	IslandPick	3,253,392	3,267,550	*nosL*, *nosY*, *nosY*, *nosD*, *nosZ、nosR*, *nirD*
	IslandPick	3,415,400	3,434,707	*narI*, *narJ*, *narH*, *narG*, *nark*, *narX*, *narL*
	IslandPick	3,745,104	3,753,063	*katE*
	IslandPick	3,878,374	3,906,751	*chrA*
	IslandPick	3,957,911	3,962,289	*arsH*, *napE*, *napD*, *napA*, *napB*, *napC*
PMCC200367	IslandPath-DIMOB	3,039,955	3,039,955	*arsC*, *arsR*, *arsB*, *arsH*
	IslandPath-DIMOB	3,065,240	3,065,240	*cusS*, *cusR*
	IslandPath-DIMOB, SIGI-HMM	6,313,982	6,348,994	*cusB*, *pcoD*, *copC*, *cusS*, *cusS*, *copA*, *copZ*, *pcoB*
	IslandPick	2,654,589	2,669,185	*narI*, *narJ*, *narH*, *narG*, *nark*, *narX*, *narL*
	IslandPick	2,838,932	2,852,956	*nosL*, *nosY*, *nosY*, *nosD*, *nosZ*, *nosR*, *nirD*
	IslandPick	2,853,008	2,869,696	*norD*, *norB*, *norC*, *norE*, *norQ*, *nirS*, *nirC*, *nirF*
	IslandPick	3,043,700	3,043,700	*arsC*, *arsR*
	IslandPick	3,763,958	3,763,958	*katE*
	IslandPick	3,922,502	3,934,847	*chrA*
	IslandPick	3,982,400	3,986,777	*arsH*, *napE*, *napD*, *napA*, *napB*, *napC*
	SIGI-HMM	3,137,393	3,156,666	*arsC*, *arsR*

### Features of the core and pan genomes

**Fig. 7:**
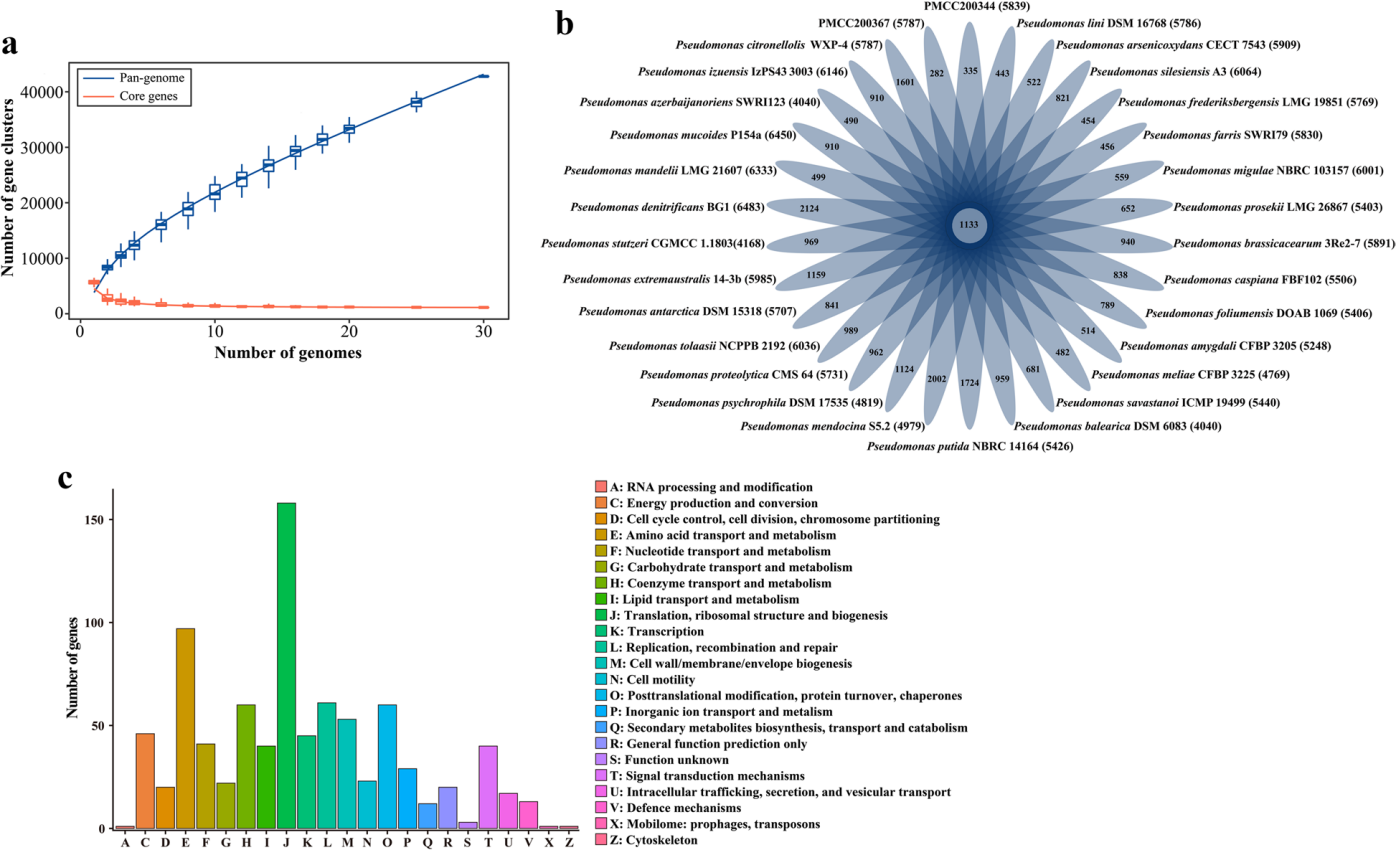
Comparison of the gene content of strains PMCC200344 and PMCC200367 with other *Pseudomonas* reference species. **a** The *Pseudomonas* core and pan genome plotted for 30 genome sequences of *Pseudomonas*-related species. **b** Venn diagram depicting the core and unique genes among PMCC200344 and PMCC200367 and 28 other relevant reference species. **c** Number of genes associated with the 23 general COG functional categories. The gene number of category B is zero

Assessment of the genetic diversity in strains PMCC200344 and PMCC200367 with other *Pseudomonas* type species was performed by comparing the *Pseudomonas* genus core and pan genomes (Fig. 7). The basic information of the strains used for pangenome analysis is indicated in Table S8. *Pseudomonas aeruginosa* PAO1 was removed from pangenome analysis due to low genome completeness (29.31%). From the alignment results, 42,773 genes were found in 30 genomes, of which 1,133 genes constituted the core genome. Strains PMCC 200344 and PMCC 200367 contained 335 and 282 unique genes, respectively. The functional categories of the core and pan genes were determined via the COG assignments among all the related species (Table S9). The results showed that the core genes presented an uneven distribution among functional categories. The core, accessory and specific genes were further analyzed, and the classification of the denitrification, cold adaptation and heavy metal resistance genes in the gene category was checked (Table S10). In the genomes of strains PMCC200344 and PMCC200367, accessory genes contained genes in the whole nitrate reduction pathway as well as a large number of genes related to cold adaptation and heavy metal resistance. However, the genes (i.e., *glnA*, *gltB* and *gltD*) related to the assimilation of ammonia nitrogen into organic nitrogen were distributed among the core genes.

### Genome collinearity analysis

**Fig. 8:**
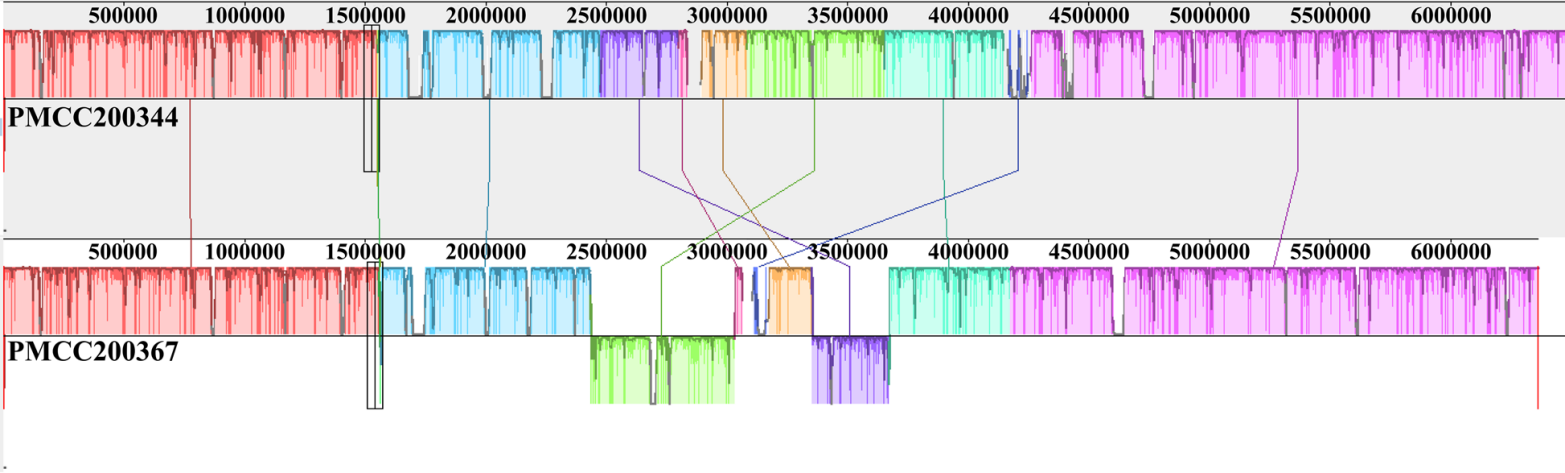
Genome collinearity analysis of *Pseudomonas* sp. PMCC200367 and PMCC200344. The different color blocks represent different conservative regions between PMCC200344 and PMCC200367, which are connected by lines of corresponding colors. The color block below the horizontal line indicates that the gene region of the same color block on the genome for comparison is inverted

Genome collinearity analysis was performed to compare the gene sequences between strains PMCC200344 and PMCC200367 at the whole-genome scale (Fig. 8). The results revealed a very high degree of genome collinearity between the two strains. There were 10 conserved regions between the two strains, accounting for 89.57% and 91.31% of the two bacterial genomes, respectively. Functional annotation aligned with the NR database showed that the genes in the noncollinear regions of the two genomes mainly belonged to mobile elements (Table S11), including IS3 family transposases, IS4 family transposases, IS66 family transposases, IS110 family transposases and IS481 family transposases.

## Discussion

Members of the genus *Pseudomonas* are known as ADB and can remove nitrogen during water treatment [1]. *P. psychrophila* RNC-1 isolated from wastewater treatment plants is effective in removing nitrate at 10 and 20 °C with the ability of assimilatory nitrate reduction and aerobic denitrification [10]. In addition, strain *P. putida* Y-9 isolated from long-term flooded paddy soil is capable of heterotrophic nitrification and aerobic denitrification at 15 °C [9]. Bacterial strains PMCC200344 and PMCC200367, which had aerobic denitrifying ability to remove nitrate in the solution at low temperature, were isolated from Arctic soils. According to the 16S rRNA gene sequence analysis, the two psychrotolerant strains were phylogenetically related to members of the genus *Pseudomonas* with sequence similarity values higher than 99.50%. In this study, according to the results of GBDP analysis as well as ANI and dDDH based on whole-genome data, PMCC200344 and PMCC200367 were verified to belong to the same species, and actually represented a novel species of the genus *Pseudomonas*. Strains PMCC200344 and PMCC200367 showed the closest relationship to *P. frederiksbergensis* LMG 19851^T^, which was isolated from soil at a coal gasification site in Denmark [18]. Strain *P. frederiksbergensis* LMG 19851^T^ is also capable of denitrification and grows at 4 and 30 °C. However, it is unclear whether strain *P. frederiksbergensis* LMG 19851^T^ has aerobic denitrification ability. The two *Pseudomonas* strains PMCC200344 and PMCC200367 can not only increase our knowledge about novel microbes in the Arctic but also provide insight into the nitrogen removal mechanism among different species of the genus *Pseudomonas* at the molecular level.

In the present study, genomic analysis of strains PMCC200344 and PMCC200367 revealed all genes of a complete set of denitrification pathways according to KEGG analysis (Fig. 5). The occurrence of metabolic pathway involves the following steps: (1) Nitrate (NO_3_^−^) and nitrite (NO_2_^−^) from outside are taken up into cells by putative nitrate/nitrite transporter (NRT); (2) nitrate entering the cell is first reduced to nitrite by nitrate reductase (NarGHJI or NapAB); (3) the resulting nitrite is then reduced to nitric oxide (NO) by nitrite reductase (NirS); (4) nitric oxide is further reduced to nitrous oxide (N_2_O) by nitric oxide reductase (NorBC); and (5) the resulting nitrous oxide is finally reduced to dinitrogen gas (N_2_) by nitrous oxide reductase (NosZ) [19]. Furthermore, putative pathways of nitrate assimilation and dissimilatory nitrate reduction to ammonium (DNRA) were also observed in the genomes of strains PMCC200344 and PMCC200367. The occurrence of metabolic pathways involves the following steps: (1) nitrite in the cell is reduced to ammonium (NH_4_^+^) by nitrite reductase (NirBD); (2) the resulting ammonium can be further incorporated into the biomolecule L-glutamine by glutamine synthetase (GlnA) and used by the bacterium [20]. Moreover, a gene involved in glutamine uptake (GlnPQ) was observed in the genomes of strains PMCC200344 and PMCC200367. The results in this study highlight that the two Arctic strains can perform simultaneous denitrification, DNRA, and nitrate assimilation under aerobic conditions. The coexistence of denitrification, DNRA and nitrate assimilation has also been observed in *Pseudomonas putida* Y-9 [21]. The heatmap (Fig. S2a) comparing the presence or absence of nitrate reduction genes across all 31 *Pseudomonas* species in this study also revealed that *P. brassicacearum* 3Re2-7, *P. citronellolis* WXP-4, *P. denitrificans* BG1, *P. extremaustralis* 14-3b, *P. farris* SWRI79^T^*, P. frederiksbergensis* LMG 19851^T^*, P. lini* DSM 16768^T^*, P. mandelii* LMG 21607^T^*, P. mucoides* P154a^T^, *P. proteolytica* CMS 64^T^, *P. silesiensis* A3^T^ and *P. stutzeri* CGMCC 1.1803^T^ possessed genes related to denitrification, DNRA and nitrate assimilation. Nitrate reduction abilities have been observed in those *Pseudomonas* species except for *P. brassicacearum* 3Re2-7, *P. denitrificans* BG1, *P. farris* SWRI79^T^ [18, 22–29]. However, different from *P. psychrophila* RNC-1 and *P. putida* Y-9 showing aerobic denitrifying ability [9, 10], genes related to nitrate reduction were not detected in the genome of type strain *P. psychrophila* DSM 17535^T^ or *P. putida* NBRC 14164^T^ (Fig. S2a). In addition, the heatmap exhibited that, there were no distinct clusters corresponding to denitrifying and non-denitrifying *Pseudomonas* strains, respectively, although strains PMCC200344 and PMCC200367 formed a cluster composed of denitrifying *Pseudomonas* strains. Nitrate-reducing bacteria *P. brassicacearum* 3Re2-7, *P. extremaustralis* 14-3b, *P. proteolytica* CMS 64^T^ and *P. stutzeri* CGMCC 1.1803^T^ did not fall into the cluster composed of strains PMCC200344 and PMCC200367. The results suggest that such multiple nitrate reduction pathways can be widely adopted by members of the genus *Pseudomonas*. Phylogenetic tree (Fig. S3a) of deduced nitrite reductase NirS sequences revealed that Arctic strains PMCC200344 and PMCC200367 showed close relationships to *P. silesiensis* A3^T^ isolated from wastewater treatment plant [25] and *P. mandelii* LMG 21607^T^ isolated from mineral water [23], different from the result of whole-genome sequence-based phylogeny analysis (Fig. 2b).

Strains PMCC200344 and PMCC200367 also revealed various genes responsible for cold adaptation, which provided the genomic basis for bacterial survival in cold environments. Cold shock proteins (Csps) can function as RNA chaperones during cold adaptation and are usually cold-induced proteins (CIPs) or cold acclimation proteins (CAPs) [30]. In this study, genes encoding CspA, CspC, CspD, CapA, and CapB were detected in the genomes of the two investigated bacteria. Furthermore, genes encoding DbpA, RhlE and DeaD were also observed in the bacterial genomes. These DEAD-box RNA helicases can increase transcription levels under low temperature stress [31]. Participating in ribosome assembly, a cold-induced protein ribosome binding factor A (RbfA) was found in the two investigated strains [31]. Compatible solutes can be accumulated by bacteria in response to environmental stresses such as salt, cold, or drought. Glycine betaine, proline betaine and trehalose are such solutes that act as effective cold stress protectants in many organisms [32–35]. In the genomes of PMCC200344 and PMCC200367, *proX*, *proW* and *proY*, which are responsible for glycine betaine transport to promote the accumulation of glycine betaine at low temperature [36, 37], were observed. Genes encoding TreX, TreY, and TreS related to trehalose biosynthesis [38] were also detected. In fact, although the 29 *Pseudomonas* reference strains are isolated from various environments worldwide, including plants, soils, desert, sewage treatment plant, mineral waters, fish, clinical specimen and Antarctic water bodies and soil, nearly all of them can grow at 4–5 °C. Therefore, it is not surprising to find that there are no distinct differences in these cold adaptation genes between the two Arctic strains and other 29 *Pseudomonas* species (Fig. S2b). It suggests that members of the genus *Pseudomonas* usually can adapt to low temperatures. Phylogenetic tree (Fig. S3b) of deduced DEAD-box RNA helicase DeaD sequences revealed that Arctic strains PMCC200344 and PMCC200367 showed close relationships to *P. arsenicoxydans* CECT 7543^T^, *P. frederiksbergensis* LMG 19851^T^ and *P. mandelii* LMG 21607^T^, different from the result of whole-genome sequence-based phylogeny analysis (Fig. 2b).

In the present study, the two bacteria were found to harbor various genes responsible for multiple heavy metal resistance, which provide a genomic basis for potential application of the strains in wastewater treatment. Metal detoxification is essential for bacterial survival in adverse environments. The chromate transporter ChrA is responsible for pumping out intracellular Cr (VI) [39], and heavy metal-transporting ATPase (ZntA) is responsible for the efflux of Pb^2+^, Zn^2+^ and Cd^2+^ from the cell [40]. The metal ABC transport system (ZnuABC) is involved in Zn^2+^ uptake [41]. The expression of *znuABC* can be reduced in response to increased zinc [42]. The membrane-bound sensor CusS and its response regulator CusR together regulate the transcription of the *cus* operon, which plays an important role in bacterial resistance to copper [43]. Furthermore, the CusCBAF complex represents an important class of bacterial efflux pumps exhibiting selectivity toward Cu (I) and Ag (I) [44]. The complex comprises the CusA transmembrane pump, the CusB soluble adaptor protein, the CusC outer-membrane pore, and the periplasmic metallochaperone CusF. In addition, the copper-binding outer membrane protein PcoB serves to permit Cu import into the periplasm as a part of an unorthodox defense mechanism against metal stress [45], and the copper resistance inner membrane protein PcoD can be responsible for copper uptake into the cytoplasm [46]. The periplasmic copper chaperone protein CopC contributes to copper resistance by binding Cu (I) and Cu (II) as a means to prevent reactivity and/or by shuttling ions between other copper resistance proteins [47]. It is interesting to find putative *afuABC*, encoding a major ferric ion transporter, in the genome sequences of the two Arctic strains. The three genes were first detected in *Actinobacillus pleuropneumoniae* [48]. In addition, the genomes of PMCC200344 and PMCC200367 contained homologs for *arsC*, *arsB*, and *arsH*, allowing the reduction of arsenate to arsenite and the pumping of arsenite out of the cell [49–51]. ArsR regulates its own expression as well as other genes in the same *ars* operon [52]. The heatmap (Fig. S2c) comparing the presence or absence of heavy metal resistance genes across all 31 *Pseudomonas* species in this study revealed that, except for *P. extremaustralis* 14-3b isolated from a water pond in Antarctica [27], *Pseudomonas* strains (including PMCC200344 and PMCC200367) containing copper resistance genes (e.g., *cusA*, *cusB* and *pcoD*) formed a cluster separate from other *Pseudomonas* species. Phylogenetic tree (Fig. S3c) of deduced arsenate reductase ArsC sequences revealed that Arctic strains PMCC200344 and PMCC200367 showed close relationships to *P. frederiksbergensis* LMG 19851^T^, similar to the result of whole-genome sequence-based phylogeny analysis (Fig. 2b). However, ArsC sequences of strains PMCC200344 and PMCC200367 formed a separate cluster distinct from the cluster containing *P. extremaustralis* 14-3b.

Genomic islands are hotspots for horizontal gene transfer (HGT) in bacteria and play an important role in bacterial evolution and adaptation. Genes that provide advantages in a selective environment have been found to be associated with genomic islands [53]. In this study, a total of 99 and 102 genomic islands were observed in strains PMCC200344 and PMCC200367, respectively. In addition to complete denitrification pathway genes, a large number of heavy metal resistance and cold adaptation genes were found to be located on genomic islands in the two Arctic bacteria. Furthermore, the results of core and pan genome analyses supported that genes involved in denitrification and DNRA and many genes related to heavy metal resistance and cold adaptation were classified as accessory genes. However, those functional genes were usually distributed on different genomic islands, suggesting that the two Arctic *Pseudomonas* strains can acquire diverse functional genes through different genomic island-mediated horizontal gene transfer among bacteria to improve their adaptability to the environment and their competitiveness.

The analysis of the core and pan genomes showed an uneven distribution among functional categories (Fig. 7c). The core genes were mainly distributed in two categories, representing translation, ribosomal structure and biogenesis (category J) and amino acid transport and metabolism (category E). Together, these genes accounted for 32.40% of the core-genome. In contrast, genes related to chromatin structure and dynamics (category B) were not found in core genes of the 30 *Pseudomonas* genomes. The uneven distribution of genes in the COG categories was mainly related to transport, metabolism and translation, suggesting that these gene functional categories enriched among the core genes reflect the adaptability and competitiveness of *Pseudomonas* spp. in complex and changeable external environments. This phenomenon can be helpful for us to understand the wide distribution of the members of the genus *Pseudomonas* in different ecological niches, including animals, plants, soil and water [54].

Belonging to the same *Pseudomonas* species, PMCC200344 and PMCC200367 showed high identity to each other. Genome collinearity analysis confirmed a very high degree of genome collinearity between the two strains. In the noncollinear regions of the two bacteria, transposase and/or integrase genes were found to randomly intersperse in the genomes (Table S11). Most of these genes were in connection with IS3, IS4, IS66, IS110 and IS481 family transposases. Insertion sequences (ISs) are the simplest type of mobile genetic elements found in prokaryotes, and typically contain only one or two open reading frames that encode genes to facilitate transposition [55]. Compared with IS630 family transposase being exclusively found in genomic islands of strain PMCC200367, IS3 and IS481 family transposases were exclusively found in strain PMCC200344 (Tables S6 and S7). In the closest relative *P. frederiksbergensis* LMG 19851^T^ capable of denitrification [18], IS4, IS5/IS1182 and IS66 family transposases were detected in bacterial genomic islands. However, IS family transposases were not observed in the genomic islands of *P. silesiensis* A3^T^, which exhibits the ability to reduce nitrate to nitrite despite the presence in the bacterial genome gene clusters coding enzymes responsible for the entire denitrification process [25]. With the ability to move through the genome as well as from one genome to another, mobile genetic elements (MGEs) play a large role in the plasticity of genomes and participate in gene acquisition [56]. No plasmid was observed in PMCC200344 and PMCC200367. In addition to prophages, integrases/transposases detected in the genome indicate that MGEs are linked to genomic plasticity, which can lead to metabolic versatility and the ability to colonize diverse environments in members of the same *Pseudomonas* species. MGEs occur in most *Pseudomonas* spp. [56–58]. More experiments should be conducted to verify the role of MGEs, including prophages and genomic islands, in the ecological function and environmental adaptation of *Pseudomonas* species in Arctic soil.

## Conclusions

In the present study, two bacterial strains PMCC200344 and PMCC200367, isolated from Arctic soil were classified and identified as novel species within the genus *Pseudomonas* based on genomic analysis as well as phenotypical characterization. Genomic analysis revealed correlations between genotype and phenotype in the two strains. Genomic islands as well as prophages were detected in the bacterial genomes, but plasmids were absent. In addition to genes involved in denitrification and DNRA, a large number of genes related to cold adaptation and heavy metal resistance were detected on genomic islands, indicating that horizontal gene transfer presented by genomic islands plays an important role in adaptability to the environment and competitiveness with other bacteria for the two strains. These analytic results not only provide insights into the genomic basis of microbial denitrification at low temperatures but also imply the potential of Arctic bacteria for aerobic denitrification in cold environments.

## Materials and methods

### Bacterial strain and DNA extraction

The strains PMCC200344 and PMCC200367 were isolated from soils under colorful snow in Arctic Ny-Alesund, Svalbard (Table S1). Based on previous 16S rRNA gene sequence analysis, both strains were identified as the genus *Pseudomonas*. A Wizard® Genomic DNA Purification Kit (Promega, Madison, WI, USA) was used to extract the genomic DNA of bacteria according to the manufacturer’s protocol.

### Phenotypic characterization

Bacterial cells were grown in R2A liquid medium (Hopebio, Qingdao, China) at 20 ℃ for 24 h and then prepared for transmission electron microscopy (TEM) using 2.5% glutaraldehyde in phosphate buffer for cell fixation. The morphology of the cells was examined using a transmission electron microscope (HT-7700, Hitachi) provided by the Institute of Hydrobiology, Chinese Academy of Sciences. Bacterial growth was detected on R2A agar (Difco, Sparks, MD, USA) at 1–35 ℃. Cell growth at 0–10% (w/v) NaCl, with a 1.0% increase, was investigated in Luria–Bertani broth prepared according to the specified formula of the medium except NaCl was not added. Cells grew at different pH values (pH 5–10 at intervals of 0.5 pH units) using a buffer system in Luria–Bertani as described previously [59]. Biochemical analysis was performed using API 20 NE (bioMérieux, Marcy-l’Étoile, France).

### Denitrifying activity assays

The BTB medium was used for preliminary screening of aerobic denitrifying bacteria [12]. Bacterial nitrate consumption was monitored by examining the blue colonies or halos formed on BTB plates. Positive strains regarded as preliminary isolated aerobic denitrifiers were then inoculated at 5% into 250-ml flasks containing 50 ml of denitrifying medium (DM). The DM medium used nitrate as the sole nitrogen source, and was composed of the following (per liter): KNO_3_, 1 g; KH_2_PO_4_, 1 g; FeCl_2_⋅6H_2_O, 0.5 g; CaCl_2_⋅7H_2_O, 0.2 g; MgSO_4_⋅7H_2_O, 1 g; sodium succinate, 8.5 g. Its pH was maintained 7.0–7.3. These flasks were incubated at 10 °C in a rotary shaker with 150 rotations per min. During 48 h of culture, the initial and final nitrate, nitrite, ammonium and total nitrogen (TN) concentrations were determined according to the standard methods [60]. The nitrate reduction capability and nitrogen removal efficiency of the bacteria were measured by calculating the amount of nitrate and TN removed.

### Genome sequencing, assembly and annotation

Genome sequencing was performed by Shanghai Majorbio Bio-pharm Technology Co., Ltd (Shanghai, China). The genome sequence was obtained via the Illumina Hiseq × 10 and PacBio RS II platforms (Table S1). The previously extracted genomic DNA was randomly fragmented through the Covaris method. Fragmented DNA was purified by the QIAquick Nucleotide Removal Kit (Qiagen, Crawley, UK). Sequencing adaptors were ligated to A-tailed 3’ ends according to the manufacturer’s instructions. A library for Illumina paired-end sequencing was prepared. For PacBio sequencing, DNA fragments were purified, end-repaired and ligated with SMRTbell sequencing adapters (Pacific Biosciences, Menlo Park, CA, USA) following the manufacturer’s recommendations. The sequencing libraries were sequenced via the combined sequencing method of Illumina Hiseq × 10 and PacBio, and each sample provided at least 100 × PacBio sequencing data and 100 × Illumina sequencing data of the genome to ensure a more complete and accurate assembly.

Retrieved raw reads were trimmed using Fastp v0.23.0 [61] to remove adapters and low quality sequences and reads. Filtered reads produced by Illumina were assembled into bacterial genomes using Unicycler v0.4.4 [62]. Reads produced by PacBio were assembled using Flye v2.5 [63]. Pilon v1.22 was used to correct potential errors in the PacBio long-reads using Illumina short-reads [64]. Coding DNA sequence (CDS) regions were predicted using Glimmer v3.02 [65]. tRNA and rRNA prediction was performed using tRNAscan-SE v2.0 [66] and Barrnap (https://github.com/tseemann/barrnap), respectively. The genomic island was predicted using IslandViewer 4 (http://www.pathogenomics.sfu.ca/islandviewer). A graphical map of the circular genome was generated using CGView v2 [67]. The predicted CDSs were annotated by querying the nonredundant (NR), Clusters of Orthologous Groups (COG), Swiss-Prot, Protein families (Pfam), evolutionary genealogy of genes: Nonsupervised Orthologous Groups (eggNOG) and Kyoto Encyclopedia of Genes and Genomes (KEGG) databases with default settings (e.g., *E* value ≤ 1e-5).

### Phylogenetic analysis

The 16S rRNA gene sequences of the two investigated strains (PMCC200344 and PMCC200367) were compared with those of the closest relatives using the Basic Local Alignment Search Tool (BLAST; http://www.ncbi.nlm.nih.gov/blast/) and EzBioCloud service (www.ezbiocloud.com). Complete genome sequences of PMCC200344 and PMCC200367 were uploaded to the Type Strain Genome Server (TYGS) for in silico based taxonomic analysis [68]. The pairwise comparison of the two strains with the type strains was performed using Genome BLAST Distance Phylogeny (GBDP) and accurate intergenomic distances inferred under the “trimming” algorithm and distance formula d0. The intergenomic distances were used to create a balanced minimum evolutionary tree using FastME 2.1.6.1 with 100 pseudobootstrap replicates for branch support. Digital DNA‒DNA hybridization (dDDH) values of the investigated strains and their closest neighbors were determined with the online server TYGS platform. Comparisons of the average nucleotide identity (ANI) were conducted using FastANI (https://github.com/ParBLiSS/FastANI).

### Comparative genomic analysis

The assembled genomes of strains PMCC200344 and PMCC200367 were compared with the available genomes of closely related *Pseudomonas* spp. deposited in the National Center for Biotechnology Information (NCBI) database. Besides seven *Pseudomonas* species with or without nitrate reduction ability, a total of 22 type strains of the genus *Pseudomonas* were selected for comparative genomics analysis. Genome sequences of the 31 *Pseudomonas* strains were uploaded to the Integrated Prokaryotes Genome and pangenome Analysis (IPGA) online server v1.09 (https://nmdc.cn/ipga/) for core and pan genomic analysis. Genes present in each selected *Pseudomonas* genome were classified as core. Software including Roary, panX, OrthoFinder, PPanGGoLiN, PanOCT, OrthoMCL, Panaroo and PPanGGoLiN were carried out with default values (i.e., identity 70, ratio (core) 0.95, and support -1). Collinearity analysis between strains PMCC200344 and PMCC200367 was performed using Mauve v2.4.0 [69]. The results were visualized with R language and ChiPlot (https://www.chiplot.online/).

### Gene functional category

The functional categories of the core gene families were analyzed and classified by the COG database. The numbers of corresponding proteins were computed for each COG term. The results were visualized by the online tool ChiPlot (https://www.chiplot.online/).

### Supplementary Information

**Additional file 1: Supplementary Table S1. **General features of *Pseudomonas* sp. PMCC200344 and PMCC200367 and MIGS mandatory information. **Supplementary Table S2. **ANI similarity between *Pseudomonas* strains PMCC200344 and PMCC200367 and the reference type strains of related species of the genus *Pseudomonas*. **Supplementary Table S3.** dDDH value between *Pseudomonas* strains PMCC200344 and PMCC200367 and the reference type strains of related species of the genus *Pseudomonas*.** Supplementary Table S4. **Genome properties and statistics of *Pseudomonas* strains PMCC200344 and PMCC200367.** Supplementary Table S5.** Basic information of genes involved in nitrate reduction pathways in bacteria using BLASTP analysis in multiple databases.** Supplementary Table S6.** Genes on the genomic islands of *Pseudomonas* strain PMCC200344.** Supplementary Table S7.** Genes on the genomic islands of *Pseudomonas* strain PMCC200367.** Supplementary Table S8.** Basic information on the *Pseudomonas* species used for pan genome analysis.** Supplementary Table** **S9.** COG annotation of core and pan genes across all 30 *Pseudomonas* species studied.** Supplementary Table S10.** Classification of the denitrification, cold adaptation and heavy metal resistance genes in the gene category.** Supplementary Table S11.** Genes in the noncollinearity region of *Pseudomonas* strains PMCC200344 and PMCC200367.** Supplementary Figure S1.** Related denitrification genes in PMCC200344 (a) and PMCC200367 (b).** Supplementary Figure S2.** Heatmaps comparing the presence or absence of nitrate reduction (a), cold adaptation (b) and heavy metal resistance (c) genes across all 31 *Pseudomonas* species studied. **Supplementary Figure S3.** Phylogenetic trees of deduced nitrite reductase NirS (a), DEAD-box RNA helicase DeaD (b) and arsenate reductase ArsC (c) sequences from Arctic strains PMCC200344 and PMCC200367 and the reference type strains of related species of the genus *Pseudomonas*. The scale bar indicates evolutionary distance.

## Data Availability

The datasets generated and analysed during the current study are available in the NCBI repository, accession numbers: CP125618 for *Pseudomonas* sp. PMCC200344, complete genome; CP125619 for *Pseudomonas* sp. PMCC200367, complete genome.

## Abbreviations

ADB: Aerobic Denitrifying Bacteria
ANI: Average Nucleotide Identity
CAPs: Cold Acclimation Proteins
CDS: Coding Sequence
CIPs: Cold-induced Proteins
COG: Clusters of Orthologous Groups
Csps: Cold shock proteins
dDDH: Digital DNA-DNA Hybridization
DNRA: Dissimilatory Nitrate Reduction to Ammonium
GBDP: Genome BLAST Distance Phylogeny
HGT: Horizontal Gene Transfer
IPGA: Integrated Prokaryotes Genome and pan-genome Analysis
KEGG: Kyoto Encyclopedia of Genes and Genomes
MGEs: Mobile Genetic Elements
NCBI: National Center for Biotechnology Information
NR: Non-Redundant
ROS: Reactive Oxygen Species
TEM: Transmission electron microscopy
TYGS: Type Strain Genome Server
