# Supplementary material for: Comparative genomic analysis of two Arctic Pseudomonas strains reveals insights into the aerobic denitrification in cold environments
Source: BMC Genomics. 2023 Sep 11;24:534. doi: 10.1186/s12864-023-09638-1 (PMC10494350; doi:10.1186/s12864-023-09638-1)
Supplement: Supplementary file 1 — Additional file 1: Supplementary Table S1. General features of Pseudomonas sp. PMCC200344 and PMCC200367 and MIGS mandatory information. Supplementary Table S2. ANI similarity between Pseudomonas strains PMCC200344 and PMCC200367 and the reference type strains of related species of the genus Pseudomonas. Supplementary Table S3. dDDH value between Pseudomonas strains PMCC200344 and PMCC200367 and the reference type strains of related species of the genus Pseudomonas. Supplementary Table S4. Genome properties and statistics of Pseudomonas strains PMCC200344 and PMCC200367. Supplementary Table S5. Basic information of genes involved in nitrate reduction pathways in bacteria using BLASTP analysis in multiple databases. Supplementary Table S6. Genes on the genomic islands of Pseudomonas strain PMCC200344. Supplementary Table S7. Genes on the genomic islands of Pseudomonas strain PMCC200367. Supplementary Table S8. Basic information on the Pseudomonas species used for pan genome analysis. Supplementary Table S9. COG annotation of core and pan genes across all 30 Pseudomonas species studied. Supplementary Table S10. Classification of the denitrification, cold adaptation and heavy metal resistance genes in the gene category. Supplementary Table S11. Genes in the noncollinearity region of Pseudomonas strains PMCC200344 and PMCC200367. Supplementary Figure S1. Related denitrification genes in PMCC200344 (a) and PMCC200367 (b). Supplementary Figure S2. Heatmaps comparing the presence or absence of nitrate reduction (a), cold adaptation (b) and heavy metal resistance (c) genes across all 31 Pseudomonas species studied. Supplementary Figure S3. Phylogenetic trees of deduced nitrite reductase NirS (a), DEAD-box RNA helicase DeaD (b) and arsenate reductase ArsC (c) sequences from Arctic strains PMCC200344 and PMCC200367 and the reference type strains of related species of the genus Pseudomonas. The scale bar indicates evolutionary distance. [file 12864_2023_9638_MOESM1_ESM.zip › Table S8..docx]

**Table S8.** Basic information on the *Pseudomonas* species used for pan genome analysis

| **Strain name** | **Accession number** | **GC%** | **Genome size (bp)** | **Isolation source** | **Nitrate reduction** |
| --- | --- | --- | --- | --- | --- |
| **PMCC200344** | CP125618 | 58.6 | 6,478,166 | Arctic soil | + |
| **PMCC200367** | CP125619 | 58.7 | 6,360,061 | Arctic soil | + |
| *Pseudomonas aeruginosa* PAO1 | AE004091 | 66.5 | 6,264,404 | Wound isolate | + |
| *Pseudomonas amygdali* CFBP 3205^T^ | NZ_JYHB00000000.1 | 58.3 | 5,719,146 | Almond, Greece | - |
| *Pseudomonas antarctica* DSM 15318^T^ | NZ_UYXQ00000000.1 | 59.5 | 6,205,254 | Freshwater, Antarctica | + |
| *Pseudomonas arsenicoxydans* CECT 7543^T^ | NZ_LT629705.1 | 58.7 | 6,502,978 | Atacama Desert, Chile | - |
| *Pseudomonas azerbaijanoriens* SWRI123^T^ | NZ_CP077078.1 | 60.1 | 6,228,218 | Rhizosphere of wheat, Iran | - |
| *Pseudomonas balearica* DSM 6083^T^ | CP007511 | 64.7 | 4,383,480 | Sewage water, Spain | + |
| *Pseudomonas brassicacearum* 3Re2-7 | CP034725 | 60.5 | 6,738,544 | Potato endorhiza | + |
| *Pseudomonas caspiana* FBF102^T^ | NZ_LOHF00000000.1 | 56.9 | 6,053,735 | Citrus orchards, Iran | - |
| *Pseudomonas citronellolis* WXP-4 | CP034688 | 67.4 | 6,672,752 | Sewage water, Hangzhou China | + |
| *Pseudomonas denitrificans* BG1 | CP043626 | 65.0 | 6,880,508 | *Prosopis cineraria* nodule | + |
| *Pseudomonas extremaustralis* 14-3b | AHIP00000000 | 60.6 | 6,587,033 | Temporary water pond, Antarctica | + |
| *Pseudomonas farris* SWRI79^T^ | NZ_JAHSTV000000000.1 | 58.7 | 6,486,948 | Wheat rhizosphere, Iran | + |
| *Pseudomonas foliumensis* DOAB 1069^T^ | NZ_JACONW000000000.1 | 57.2 | 5,962,112 | Wheat leaf, Alberta Canada | - |
| *Pseudomonas frederiksbergensis* LMG 19851^T^ | NZ_FNTF00000000.1 | 58.9 | 6,394,336 | Soil, Denmark | + |
| *Pseudomonas izuensis* IzPS43 3003^T^ | NZ_WTFT00000000.1 | 59.6 | 6,865,466 | Soil, Japan | - |
| *Pseudomonas lini* DSM 16768^T^ | NZ_JYLB00000000.1 | 58.8 | 6,495,504 | Bulk and rhizospheric soil, France | + |
| *Pseudomonas mandelii* LMG 21607^T^ | NZ_LT629796.1 | 59.2 | 7,041,758 | Mineral water, France | + |
| *Pseudomonas meliae* CFBP 3225^T^ | NZ_JYHE00000000.1 | 58.4 | 5,143,641 | *Melia azedarach* | - |
| *Pseudomonas mendocina* S5.2 | AMCD01000000 | 62.4 | 5,351,183 | France vineyard soil | + |
| *Pseudomonas migulae* NBRC 103157^T^ | NZ_BDAG00000000.1 | 59.1 | 6,667,355 | Mineral waters | - |
| *Pseudomonas mucoides* P154a^T^ | NZ_JACOPW000000000.1 | 58.8 | 7,040,872 | Rainbow trout, Turkey | + |
| *Pseudomonas prosekii* LMG 26867^T^ | LT629762 | 59.6 | 6,092,055 | Soil, Antarctica | - |
| *Pseudomonas proteolytica* CMS 64^T^ | VFEV00000000 | 60.5 | 6,350,040 | Cyanobacterial mat, Antarctica | + |
| *Pseudomonas psychrophila* DSM 17535^T^ | JYKZ00000000 | 57.5 | 5,334,010 | Coldroom for food storage, Japan | - |
| *Pseudomonas putida* NBRC 14164^T^ | AP013070 | 62.3 | 6,156,701 | Soil | - |
| *Pseudomonas savastanoi* ICMP 19499 | NZ_LKCI00000000.1 | 58.3 | 6,162,306 | Kiwi fruit, New Zealand | - |
| *Pseudomonas silesiensis* A3^T^ | NZ_CP014870.1 | 59.6 | 6,823,539 | Wastewater treatment plant, Poland | + |
| *Pseudomonas stutzeri* CGMCC 1.1803^T^ | CP002881 | 63.9 | 4,547,930 | Clinical specimen | + |
| *Pseudomonas tolaasii* NCPPB 2192^T^ | PHHD01000001 | 60.5 | 6,792,797 | *Agaricus bisporus*, UK | - |

+: Positive; -: Negative.
